# Essential newborn care practice and associated factors among health care providers in Northeast Ethiopia: a cross-sectional study

**DOI:** 10.1186/s13690-021-00613-4

**Published:** 2021-06-01

**Authors:** Gebrehana Ashenef, Akine Eshete, Betregiorgis Zegeye, Tadesse Tsehay Tarekegn, Mitku Mammo Taderegew

**Affiliations:** 1 Department of Public Health, Debre Berhan Health Science College, Debre Berhan, Ethiopia; 2 Department of Public Health, College of Health Science, Debre Berhan University, Debre Berhan, Ethiopia; 3 Shewarobit Field Office, HaSET Maternal and Child Health Research Program, Addis Ababa, Ethiopia; 4 Department of Nursing, College of Medicine and Health Sciences, Wolkite University, Wolkite, Ethiopia; 5 Department of Biomedical Sciences, College of Medicine and Health Sciences, Wolkite University, P.O. Box 07, Wolkite, Ethiopia

**Keywords:** Practice, Essential newborn care, Health care providers, Ethiopia

## Abstract

**Background:**

Globally, 2.7 million children die during the neonatal period annually. Ethiopia is one of the ten countries with the highest number of neonatal deaths. The practice of poor essential newborn care contributes to the problem. Hence the study was conducted to assess the essential newborn care practice and associated factors among health care providers from selected health facilities in Northeast Ethiopia.

**Methods:**

Facility-based cross-sectional study was conducted among health care providers working in selected health facilities in Northeast Ethiopia from February-25 to March-25, 2019. Data were collected by a pre-tested questionnaire and an observational checklist. Then data were edited into Epi-data-7.2.0.1 and analyzed by using SPSS-25 software. The degree of association was assessed using binary logistic regression analysis. *P*-value < 0.05 was considered statistically significant.

**Results:**

A total of 256 health care providers were included in the study. Overall, 62.9% (95%CI: 57.0–68.8%), and 73.8% (95%CI: 68.4–79.2%) of the health care providers had adequate knowledge and good practice on essential newborn care activities, respectively. The presence of supportive supervision (AOR = 2.09, 95%CI = 1.07–4.11), the interest of health care providers to work at delivery room (AOR = 1.97, 95%CI = 1.00–3.88), and availability of vitamin-K (AOR = 4.81, 95%CI = 1.07–21.64) were significantly associated with essential newborn care practices.

**Conclusions:**

A significant number of health care providers had inadequate knowledge and poor practice of essential newborn care. Availability of vitamin-K, the interest of the health care providers to work in the delivery room and the presence of supportive supervision were the factors affecting essential newborn care practice. Hence, giving in-service training, supportive supervision, and providing supplies should be strengthened to enhance essential newborn care activities.

**Supplementary Information:**

The online version contains supplementary material available at 10.1186/s13690-021-00613-4.

## Background

Globally, an estimated 2.5 million neonates die every year, approximately 7000 deaths every day. The majority of all neonatal deaths (75%) occur during the first week of life, and close to one quarter dying within the first 24 h of life. Approximately 98% of neonatal deaths occur in developing regions [1, 2].

The risk of neonatal deaths is highest in Africa (41 deaths per 1000 live births). The sub-Saharan regions of Eastern, Western, and Central Africa have between 42 and 49 neonatal deaths per 1000 live births. Ten countries account for 67% of the total newborn deaths from the global total neonatal deaths. Ethiopia is ranked at the sixth among ten countries with the highest number of neonatal deaths [3, 4].

According to the Ethiopian Demographic and Health Survey-2019, one in every 34 children dies within the first month, one in every 23 children dies before celebrating the first birthday, and one in every 18 children dies before reaching the fifth birthday. Thus, neonatal deaths account for more than 70% of infants’ mortality and about 55% of under-five mortality [5].

Most of the newborn deaths were due to preventable or treatable causes that occur because of poor implementation of essential newborn care (ENBC), and up to two-thirds could be saved by providing essential care to the newborns [6, 7]. As delivery and the first few hours of life are a critical period for the further growth and development of infants, the future well-being of the child will be determined by the quality of care that the newborn receive. Evidence showed that children who die within the first 28 days of birth suffer from conditions and diseases associated with a lack of quality of care at birth or skilled care and treatment immediately after birth and on the first day of life [2, 8].

Provision of immediate newborn care will prevent many newborn emergencies, meanwhile it can prevent 50% of complications and 45% of neonatal deaths which occur during birth and at postnatal periods [8–10]. Despite these, relatively little is known about the level of ENBC service delivery by health care providers and associated factors in Ethiopia particularly in the study area.

Thus, the finding of the study will provide baseline information on ENBC practice and its related factors which are helpful for policymakers, and other stakeholders to develop appropriate interventions towards improving newborns’ health care delivery. The results will also benefit other researchers, as baseline data for further investigation. Hence the study was conducted to assess the practice of ENBC and associated factors among health care providers (Midwives, Nurse, General practitioners, Emergency surgeons, and Public health officers) in the health facilities of Northeast Ethiopia.

## Methods

### Study design, and setting

A facility-based cross-sectional study was conducted among health care providers working in the selected health facilities in the North Shoa administrative zone of Northeast Ethiopia from February-25-2019 to March-25-2019. North Shoa administrative zone is situated 130 km northeast of Addis Ababa, the capital city of Ethiopia. The administrative zone has 24 districts with a total of 9 government hospitals and 79 health centers (HC).

The study populations were all selected health care providers working in the selected hospitals and HC during the study periods. All the health care providers who were working in maternal and child health (MCH) unit at selected health facilities were eligible for the study, whereas those health care providers who were assigned in MCH units but not participate in immediate newborn care activities during the study periods were excluded from the study.

### Sample size determination

The required sample size of the study was estimated using a single population proportion formula, by considering: *P* = 81.1% (expected prevalence of essential newborns care practice among health care providers) [11], 95%CI (Za/_2_ = 1.96), and 5% margin of error. Then the minimum sample size obtained was 235. After adding a 10% non-response rate, a total of 259 health care providers were included in the study.

From all health facilities found in the district, six hospitals and 24 HC were randomly selected using a lottery method. Then, the number of health care providers who were selected from each selected health facility was determined proportionally. Finally, a convenient sampling method was used to select each study participant in each selected health facility.

### Data collection procedures

Data were collected using a semi-structured questionnaire and an observational checklist. The questionnaire and checklists were adopted from different literature with modifications for face-to-face interviews and clinical observation (Additional file 1: Table S1). The questionnaire contains variables for assessing socio-demographic characteristics, availability of supplies, and knowledge of health care providers towards essential newborn care. The practices of health care providers on essential newborn care were assessed by using an observational checklist while the health care provider performing newborn cares. .

Two data collectors and one supervisor from the non-selected hospitals and health centers were recruited for each selected health facility. After obtaining informed consent from each participant, information regarding sociodemographic characteristics, availability of supplies during their practice, knowledge, and practice of the participant towards ENBC were collected by data collectors.

To maintain the quality of the data, training was given for the data collectors and supervisors. The questionnaire was also pre-tested on 5% of the total sample size in a separate health facility. The questionnaire and data collection procedures were checked frequently for its consistency and completeness by the investigators and supervisor.

### Operational definitions

#### Knowledge

Participants were considered as having good knowledge of essential newborn care if they scored greater than or equal to the mean score (8) of 16 knowledge-related questions. If they score below the mean score considered as having poor knowledge.

#### Practice

Refers to the practice of health care providers based on the prepared checklist regarding essential new-born care activities. Participants were considered as having good practice if they correctly perform at least 70% or above the tasks in the checklists. If they perform below 70% of the tasks in the checklist considered as having poor practice.

#### Clean delivery room

Separated room with at least one newborn care area for every 4 labor tables, and all surfaces were cleaned every morning and all equipment and surfaces were cleaned after every delivery [12].

### Statistical analysis

Data were checked for completeness and consistency. Then data were entered into Epi-data-7.2.0.1 then analyzed by using SPSS-25 software. Categorical variables were stated in frequency using numbers (percentage) whereas the continuous variables were stated using means ± standard deviation (SD). Then the data were processed by using frequency distribution, and cross-tabulation. Binary logistic regression analysis was used to identify factors associated with the practice of essential newborns care among health care providers. All independent variables with a *p*-value < 0.20 in the bivariate analysis were entered into a multivariable regression model to control the effect of a confounder. The degree of association was measured by using the odds ratio (ORs) with 95% CI. *P*-value with < 0.05 was considered statistically significant.

## Results

### Socio-demographic characteristics of the study participants

A total of 256 health care providers (with a response rate of 98.8%) were recruited in the study, of which 153 (59.8%) were males. Nearly half of the participants, 123 (48.0%) were between the age of 25–29 years. The majority of participants, (89.5%) were orthodox Christian. From the total of the study participants, 105 (41.0%) were midwives and 96 (37.5%) were nurse. One hundred seventy-nine (69.9%) of the participants were working at the health center, 56 (21.9%) were working at District hospitals and 144 (56.3%) of them were interested to work in the delivery room (Table 1).

**Table 1:** Socio-demographic characteristics of the study participants at Northeast Ethiopia, 2019 (*N* = 256)

Variable	Frequency (*n* = 256)	Percentage (%)
Age (years)		
< 25	46	18.0
25–29	123	48.0
30–34	52	20.3
≥ 35	35	13.7
Marital Status		
Married	130	50.8
Single	116	45.3
Divorced	10	3.9
Religion		
Orthodox	229	89.4
Muslim	13	5.1
Protestant	14	5.5
Educational status		
Diploma	126	49.2
Degree	111	43.4
Master	13	5.1
Doctor	6	2.3
Employment status		
Midwife	104	40.6
Nurse	97	37.9
Doctor	7	2.7
Emergency surgeon	14	5.5
Health officer	34	13.3
Work experience (years)		
< 5	145	56.6
6–10	71	27.7
11–15	25	9.8
> 15	15	5.9
Monthly income (ETB)		
< 3105 birr	45	17.6
3105–3825 birr	64	25.0
3826–4545 birr	53	20.7
> 4546	94	36.7

*ETB* Ethiopian birr

### Assessment of availabilities of supplies and drugs

The majority (85.5%) of participants stated that all the necessary supplies were available for running ENBC, and 156 (60.9%) got supervision given by other health care providers or stakeholders as needed. The majority of the health care providers 183 (71.5%) were not trained on basic ENBC but 216 (84.4%) health care providers said that training guidelines of ENBC are available at their health facilities. The majority (95.7%) of the health care providers said that Vitamin K injection is available at their health facility (Table 2).

**Table 2:** Assessment of different supplies and drugs available in the health facility for ENBC, practice in Northeast Ethiopia, 2019 *(N* = 256)

Variables	Yes (%)	No (%)
Are there different supplies and drugs being available in this health facility to provide ENBC?	219 (85.5)	37 (14.5)
Is there any internal or external supportive supervision regarding to ENBC?	156 (60.9)	100 (39.1)
Is there clean delivery room?	248 (96.9)	8 (3.1)
Availability of prepared cord tie	252 (98.4)	4 (1.6)
Is baby identification material prepared and available	241 (94.1)	15 (5.9)
Is suction device available in the health facility	252 (98.4)	4 (1.6)
Are there training guidelines available?	216 (84.4)	40 (15.6)
Vitamin K injection	245 (95.7)	11 (4.3)
TTC eye ointment	253 (98.8)	3 (1.2)
Trained about ENBC	62 (24.2)	194 (75.8)

*ENBC* Essential newborns care, *TTC* Tetracycline eye ointment

### Assessment of the health care provider’s knowledge regarding ENBC

The majority (92.6%) of the study participants stated that TTC eye ointment should be applied within 1 h of delivery, and 252 (98.4%) of them said breastfeeding should be initiated within 1 h of delivery. About 251 (98%) of the health care providers knew that colostrum helps to prevent newborn babies from infection.

The majority (98.8%) of the study participants knew that skin-to-skin contact of the newborn with the mother helps to prevent hypothermia and 210 (82.0%) said that newborns should not be bathed immediately even if the baby has stained meconium. Overall, 62.9% of participants had a good knowledge of essential newborns care practice (Table 3).

**Table 3:** Knowledge of health professionals towards steps of immediate newborn care at different health facilities in Northeast Ethiopia, 2019 (*N* = 256)

Variables	Yes (%)	No (%)
Is TTC eye ointment should be applied within 1 h of delivery?	237 (92.6)	19 (7.4)
Should not the neonates be bathed immediately after birth if blood or meconium is stained on the skin?	210 (82.0)	46 (18.0)
Is initiating breast feeding important for child if started within 1 h?	252 (98.4)	4 (1.6)
Should the neonates be referred when they develop complications immediately after birth?	243 (94.9)	13 (5.1)
Does skin-to-skin contact prevent the newborns from hypothermia and help to stay warm?	253 (98.8)	3 (1.2)
Is the newborn should be placed on the mother’s abdomen immediately after birth?	244 (95.3)	12 (4.7)
Is administering Vit K important to prevent bleeding?	243 (94.9)	13 (5.1)
Is unable to suck or cry a newborn danger sign?	242 (94.5)	14 (5.5)
All babies should be assessed after birth?	248 (96.9)	8 (3.1)
Should you clean the face and eye of baby when the head of the neonate is delivered?	249 (97.3)	7 (2.7)
Should the mothers be assessed after the delivery while she is on delivery coach?	245 (95.7)	11 (4.3)
Is the colostrum prevents the neonates from infection and gives important nutrient?	251 (98.0)	5 (2.0)
Is placing identification band on wrists or ankles mandatory?	226 (88.3)	30 (11.7)
A newborn child should be bathed after 24 h even if the neonate has stained meconium?	239 (93.4)	17 (6.6)
Are newborns should always be dry after birth?	247 (96.5)	9 (3.5)
Are newborns should always be weighed after birth?	253 (98.8)	3 (1.2)

*TTC* Tetracycline eye ointment

### Assessment of essential newborns care practice of the study participants

In this study, the overall practice of ENBC was 73.8% (95%CI: 68.4–79.2). Almost all the participants were applying cleaning the face and eye of the baby when the head is delivered. The majority (92.2%) of them have checked the breathing of the baby and assesses the color of the newborn immediately after delivery. All of them apply to place the baby on the mother’s chest for skin-to-skin contact and initiate immediate breastfeeding. However, the majority of participants (82.0%) didn’t apply Chlorhexidine gel on the cord within 30 min of delivery (Table 4).

**Table 4:** Practice of health care providers on immediate newborn care at different health facilities in Northeast Ethiopia, 2019 (*N* = 256)

Variable	Tasks performed (%)	Tasks not performed (%)
Placed on the mother’s abdomen, dry and stimulate neonate within 30 s after a vaginal delivery.	254 (99.2)	2 (0.8)
Assess breathing and color after 30 s up to 1 min	236 (92.2)	20 (7.8)
The cord was clamped within 1–3 min when the baby does cry or breaths well. When the breaths was < 30 per minute, blue tongue, lips or trunk or gasping then resuscitating was immediately started.	256 (100.0)	0 (0.0)
Chlorhexidine gel (4%) was applied on the cord within 30 min of delivery	46 (18.0)	210 (82.0)
Place the infant in skin-to-skin contact on the mother’s chest and cover both with clean linen and blanket as required.	256 (100.0)	0 (0.0)
Initiate breastfeeding immediately within 1 h	256 (100.0)	0.0%
Eye care-Apply tetracycline eye ointment within 90 min of delivery	254 (99.2)	2 (0.8)
Give vitamin K, 1 mg IM on anterior mid-lateral thigh (within 90 min)	251 (98.0)	5 (2.0)
Place the baby identification bands on the wrist and ankle (within 90 min)	188 (73.4)	68 (26.6)
Weigh the newborn within 90 min & when babies is stable and record all care given	254 (99.2)	2 (0.8)

*IM* Intramuscular

### Factors associated with the practice of essential newborns care

In bivariate logistic regression analysis, age of participants, monthly income, work experience, the interest of the health care providers to work in the delivery room, type of the health facility, supportive supervision, availability of vitamin K, and training of health care providers on ENBC had *p*-value < 0.20 and candidate in multivariable logistic regression analysis.

In multivariable logistic regression analysis, the odds of practicing ENBC among participants who had got supportive supervision on ENBC was two (AOR = 2.09, 95%CI = 1.07–4.11) times higher than those who hadn’t. Participants who were interested to work in the delivery room were also nearly 2 times (AOR = 1.97, 95%CI = 1.00–3.88) more likely to have a good practice of ENBC. Furthermore, participants working in the health facility with the availability of Vitamin K were nearly five (AOR = 4.81, 95%CI = 1.07–21.64) times more likely to have a good practice of ENBC (Table 5).

**Table 5:** Multivariate analysis of factors associated with essential newborn care practice among health care providers in North shoa zone, Northeast Ethiopia, 2019 (*N*=256)

Variable	Practice		COR (95% CI)	AOR (95% CI)
	Good (%)	Poor (%)		
Monthly income (ETB)				
<3105	35(77.8)	10(22.2)	1	1
3105-3825	47(73.4)	17(26.6)	0.79 (0.32-4.93)	1.28(0.46-3.62)
3826-4545	33(62.3)	20(37.7)	0.47(0.19-1.16)	1.03(0.33-3.27)
>4546	74 (78.7)	20(21.3)	1.06(0.49-2.95)	2.15(0.65-7.12)
Work experience (years)				
<5	112(77.2)	33(22.8)	1	1
6-10	50(70.4)	21(29.6)	0.70(0.37-1.33)	0.72(0.30-1.71)
11-15	21(84.0)	4(16.0)	1.55(0.49-4.83)	1.75(0.23-13.28)
>15	6(40.0)	9(60.0)	0.19(0.65-0.59) *	1.44(0.08-25.57)
Interest to work in labor room				
No	72(64.3)	40(35.7)	1	1
Yes	117(81.3)	27(18.7)	2.41(1.36-4.26) *	1.97(1.00-3.88) *
Health facility				
Referral hospital	17(80.9)	4(19.1)	1	1
District hospital	51(91.1)	5(8.9)	2.40(0.58-9.98)	2.86(0.58-14.23)
Health center	121(67.6)	58(32.4)	0.49(0.16-1.53)	1.03(0.28-13.80)
Presence of supervision				
Absent	65(65.0)	35(35.0)	1	1
Present	124(79.5)	32(20.5)	2.09(1.26-3.90) *	2.09(1.07-4.11) *
Availability of Vitamin K				
No	3(27.3)	8(72.7)	1	1
Yes	186(75.9)	59(24.1)	8.41(1.16-32.72) *	4.81(1.07-9.64) *
Age of health care providers (years)				
<25	38(82.6)	8(17.4)	1	1
25-29	84(68.3)	39(31.7)	0.45(0.19-1.06)	0.59(0.23-1.60)
30-34	46(88.5)	6(11.5)	1.61(0.52-5.06)	1.56(0.38-6.39)
≥35	21(60.0)	14(40.0)	0.32(0.02-0.58) *	0.06(0.04-1.07)
Training on ENBC				
No	131(71.6)	52(28.4)	1	
Yes	58(79.5)	15(20.5)	1.53(1.03-4.55) *	1.52(0.65-3.54)

Note (N=sample size; * *p*-value <0.05; *Abbreviation: AOR* Adjusted odd ratio, *COR* Crude odd ratio, *D* District, *ETB* Ethiopian birr

## Discussion

In this institutional-based cross-sectional study, essential newborns care practice and its predictor among health care providers in northeast Ethiopia has been assessed. It was found that good practice of essential newborns care among health care providers was 73.8% (95%CI = 68.4–79.2). It was also found that the practice of essential newborns care was significantly associated with the presence of supportive supervision, availability of vitamin K, and participants’ interest to work in the delivery room.

The proportion of practice of ENBC in this study was almost similar to the study conducted at Tigray, northern Ethiopia (72.8%) [13]. This similarity might be because the study in the Tigray region was recently conducted, both the region might have taken recently updated training on ENBC. However, in this study, the practice of the ENBC was higher than the study conducted in Sudan (58.9%) [10], and Bahir Dar, Northwest Ethiopia (59.7%) [14]. On the other hand, the practice of ENBC in this study was lower than the study conducted at Eastern Tigray (92.9%) [15], and Addis Ababa, the capital city of Ethiopia (81%) [16]. The possible explanation for these variations in the prevalence of essential newborns care practice might be due to the difference in the study setting, a possible slight difference in the data collection tools used, and the difference in the educational qualification of the study participants.

In this study, supportive supervision given for health care providers was significantly associated with the practice of ENBC which is similar to the study conducted in Jimma, Southwest Ethiopia [17] and Tigray, Northern Ethiopia [18] but unlike the study conducted in Jimma and Tigray in which knowledge and educational level were the determinants of ENBC, in this study knowledge and educational level were not significantly associated with the practice of ENBC. This difference might be due to the variations of the number of health care providers who received in-service training during supportive supervision.

In this study availability of vitamin K was significantly associated with the practice of ENBC but there was no significant association with the type of health facility. In the study conducted in the Tigray region besides the availability of materials, the type of health facility was significantly associated with the practice of ENBC [19]. This difference might be due to variation in in-service training and supportive supervision which was given for health care providers on ENBC in HC and hospitals. This increase in the practice of ENBC among health care providers in the health facilities with the availability of vitamin K may be an indication that the health facilities’ preparedness for applying ENBC practice according to the recommended standards.

The interest of the health care providers to work in the delivery room was also significantly affecting the practice of essential newborn activities. Participants who were interested to work in the delivery room were nearly two times more likely to have a good practice of ENBC. This is similar to the study findings conducted in Jimma [17], and Wolaita zone [20]. This might be because health care providers who were interested to work in the delivery room may get more information and knowledge on areas where they are interested to work which can lead to an increment of the level of practice.

## Conclusion

A significant number of health care providers had inadequate knowledge and poor practice on ENBC. Availability of vitamin K, the health care providers’ interest to work in the delivery room, and the presence of supportive supervision were the factors affecting ENBC. Hence, improving in-service training, supportive supervision, and providing supplies should strengthen to enhance ENBC activities.

### Limitations of the study

Due to the cross-sectional nature of the study temporal and cause-effect relationship was not identified, and the study was not considering private health institutions were the limitations of the study.

## Supplementary Information

**Additional file 1: Table S1**: English version questionnaire on essential newborn care practice and associated factors of among health care providers in Northeast Ethiopia: A Cross-Sectional Study.

## Data Availability

The datasets used and/or analyzed during this study are available from the corresponding author on reasonable request.

## Abbreviations

AOR: Adjusted Odd Ratio
CI: Confidence interval
COR: Crude Odd Ratio
ENBC: Essential newborn care
HC: Health Center
MCH: Maternal, newborn and child health
SPSS: Statistical Package for Social Science
TTC: Tetracycline eye ointment
WHO: World Health Organization
