# Supplementary material for: Essential newborn care practice and associated factors among health care providers in Northeast Ethiopia: a cross-sectional study
Source: Arch Public Health. 2021 Jun 1;79:90. doi: 10.1186/s13690-021-00613-4 (PMC8167947; doi:10.1186/s13690-021-00613-4)
Supplement: Supplementary file 1 — Additional file 1: Table S1: English version questionnaire on essential newborn care practice and associated factors of among health care providers in Northeast Ethiopia: A Cross-Sectional Study. [file 13690_2021_613_MOESM1_ESM.docx]

**English version Questionnaire**

**Table S1**: Essential newborn care Practice and associated factors of among health care providers in Northeast Ethiopia: A Cross-Sectional Study

**Instructions:** Follow the instructions and guides under the questions to fill the questionnaire.

**Part 1: Socio – demographic characteristics**

| S.No | | Question | Response |
| --- | --- | --- | --- |
| 001 | | How old are you? Age (in completed year) | _______________ |
| 002 | | Sex of health care provider | 1. Male 2. Female |
| 003 | | What is your current marital status? | 1. Single 2. Married 3. Divorced 4. Widowed |
| 004 | | What is your religion? | 1. Orthodox 2. Muslim 3. Protestant 4. Other specify __________________ |
| 005 | | Level of education | 1. Diploma 2. Degree 3. Masters 4. Doctors |
| 006 | | Profession | 1. Midwifery 2. Nurse 3. Health officer 4. Emergency surgeon |
| 007 | | Work experience (years) | __________________ |
| 008 | | Your monthly income (ETB) | ___________ |
| 009 | | Have an interest to work in delivery room? | 1. Yes 2. No |
| 010 | | Type of health facility that you now work | 1. Referral hospital 2. District hospital 3. Health center |
| 011 | | What is your Ethnicity? | 1. Oromo 2. Amhara 3. Others, Specify______________ |
| 012 | | What is the average monthly income of the household? | ______________in birr |
| 013 | | Is there different materials or supplies-available in the health facility? | 1. Yes 2. No |
| 014 | | Is there any internal/external supportive supervision given for you? | 1. Yes 2. No if no go to Q no 016 |
| 015 | | If yes how many supportive supervision do you have get? | 1. Every month 2. Every 2-6 month 3. Every 7-12 month 4. As needed |
| **Part II questions related availability of different supplies and cleanness of health facilities (with the aid of observation)** | | | |
| 016 | | Is there clean delivery room clean? | 1. Yes 2. No |
| 017 | | Is there already prepared cord tie? | 1. Yes 2. No |
| 018 | | Is there prepared baby identification materials? | 1. Yes 2. No |
| 019 | | Is there prepared suction device? | 1. Yes 2. No |
| 020 | | Is there training guidelines available? | 1. Yes 2. No |
| 021 | | Is Vit K injection available? | 1. Yes 2. No |
| 022 | | Is TTC eye ointment available? | 1. Yes 2. No |
| 023 | | Have you got ENC training? | 1. Yes 2. No |
| 024 | | Were you counseled/advised about the importance of infant nutrition including breast feeding in your PNC follow-up visits? | 1. Yes 2. No |
| **Part III: Knowledge about essential newborn care practice among health care providers** | | | |
| 025 | Did the TTC eye ointment should be applied within one hour? | | 1. Yes 2. No |
| 026 | Did the neonate shall not bathed immediately? | | 1. Yes 2. No |
| 027 | Is initiating breast feeding important for child if started within one hour? | | 1. Yes 2. No |
| 028 | Is referral of the infant necessary when infant developing complications immediately after birth? | | 1. Yes 2. No |
| 029 | Is skin-to-skin contact of newborn neonate with mother prevent hypothermia and help baby stay warm? | | 1. Yes 2. No |
| 030 | Is the newborn child should place on the mother’s abdomen immediately? | | 1. Yes 2. No |
| 031 | Is administering Vit K is important to prevent bleeding? | | 1. Yes 2. No |
| 032 | Is it unable suck or cry is newborn danger sign? | | 1. Yes 2. No |
| 033 | All babies should be assessed after birth? | | 1. Yes 2. No |
| 034 | Is cleaning the face and the eye of baby necessary after the head is delivered? | | 1. Yes 2. No |
| 035 | Is it necessary to assess mothers after the delivery immediately on while she is on delivery coach? | | 1. Yes 2. No |
| 036 | Is the colostrum prevents from infection and gives important nutrient? | | 1. Yes 2. No |
| 037 | Is it placing identification bands on wrist or ankles are mandatory? | | 1. Yes 2. No |
| 038 | Is newborn child should be bathed after 24 hrs even if the neonate has stained meconium? | | 1. Yes 2. No. |
| 039 | Is drying the baby after birth always important? | | 1. Yes 2. No |
| 040 | Is weighing the newborn neonate after birth is always necessary? | | 1. Yes 2. No |
| **Part IV: Clinical observational checklist to assess practice of newborn care** | | | |
| 041 | Placing immediately on the mother’s abdomen after a vaginal delivery, dry and stimulate neonate within 30 seconds | | 1. Task performed 2. Task not performed |
| 042 | Assess breathing and color after 30 seconds up to 1 minute | | 1. Task performed 2. Not performed |
| 043 | If the baby does cry or breaths well clamp/tie and cut the cord within 1-3 minutes. If < 30 breaths per minute, blue tongue, lips or trunk or if gasping then start resuscitating | | 1. Task performed 2. Task not performed |
| 044 | Apply Chlorhexidine gel (4%) on the cord within 30min of delivery | | 1. Task performed 2. Task not performed |
| 045 | Place the infant in skin-to-skin contact on the mother’s chest and cover both with clean linen and blanket as required. | | 1. Task performed 2. Task not performed |
| 046 | Initiate breastfeeding immediately within 1 hour | | 1. Task performed 2. Task not performed |
| 047 | Eye care-Apply tetracycline eye ointment within 90 min of delivery | | 1. Task performed 2. Task not performed |
| 048 | Give vitamin K, 1mg IM on anterior mid-lateral thigh (within 90min) | | 1. Task performed 2. Task not performed |
| 049 | Place the baby identification bands on the wrist and ankle (within 90 min) | | 1. Task performed 2. Task not performed |
| 050 | Weigh the newborn within 90 min & when babies is stable and record all care given | | 1. Task performed 2. Task not performed |

Thank you!!!
